# Supplementary figures and images for: Pyvolve: A Flexible Python Module for Simulating Sequences along Phylogenies
Source: PLoS One. 2015 Sep 23;10(9):e0139047. doi: 10.1371/journal.pone.0139047 (PMC4580465; doi:10.1371/journal.pone.0139047)

**Observed dN/dS**

0.5

0.4

0.3

0.2

0.1

0.1

0.2

0.3

0.4

0.5

**Simulated dN/dS**

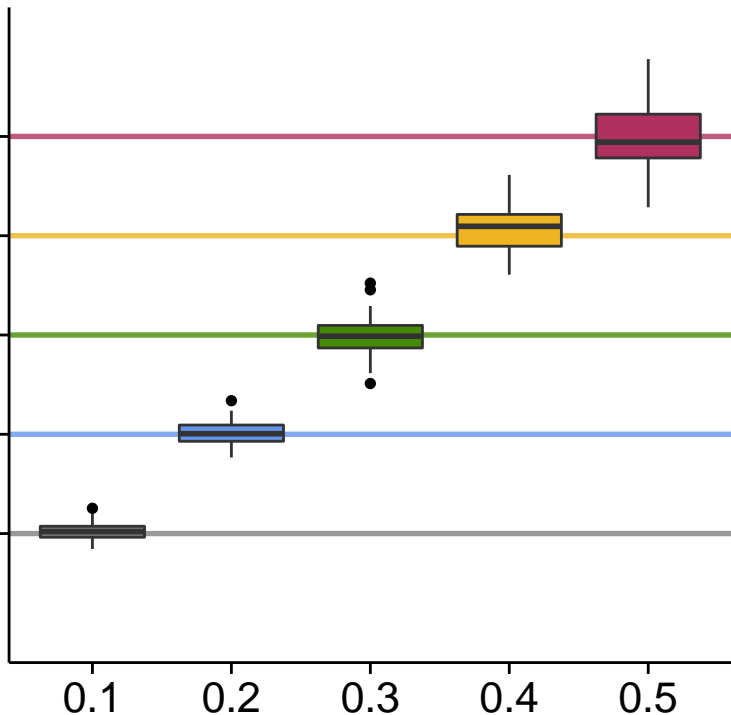

Supplement: S1 File — (ZIP) [file pone.0139047.s001.zip › SI/plot/validated_branchhet.pdf]

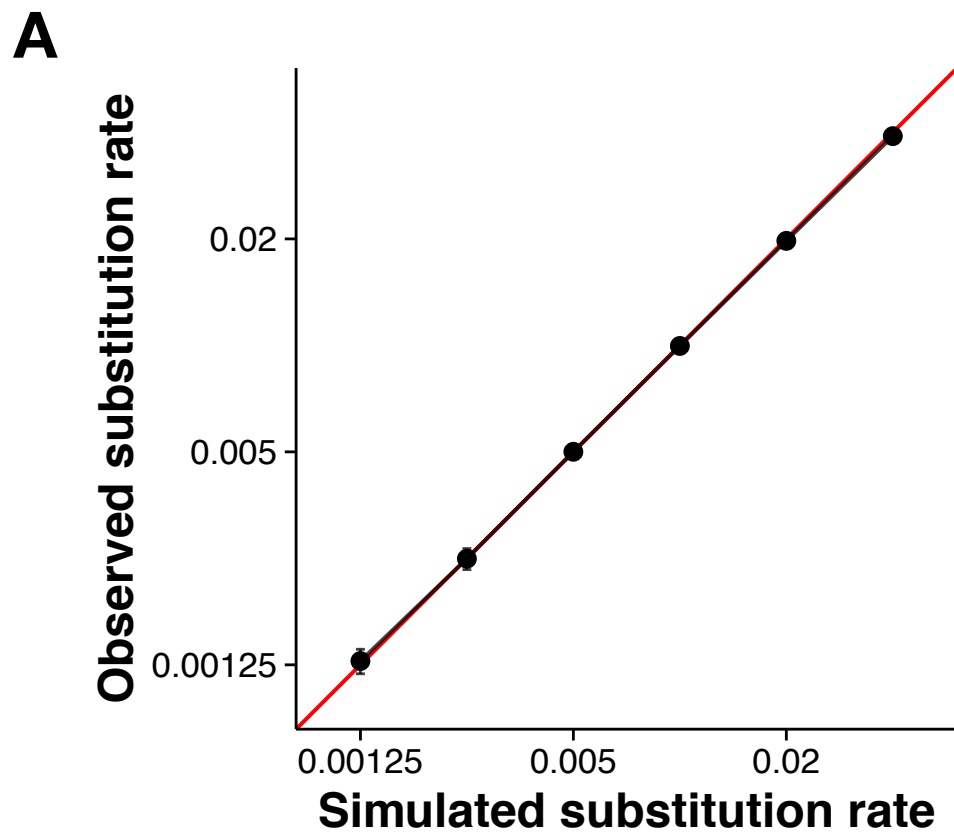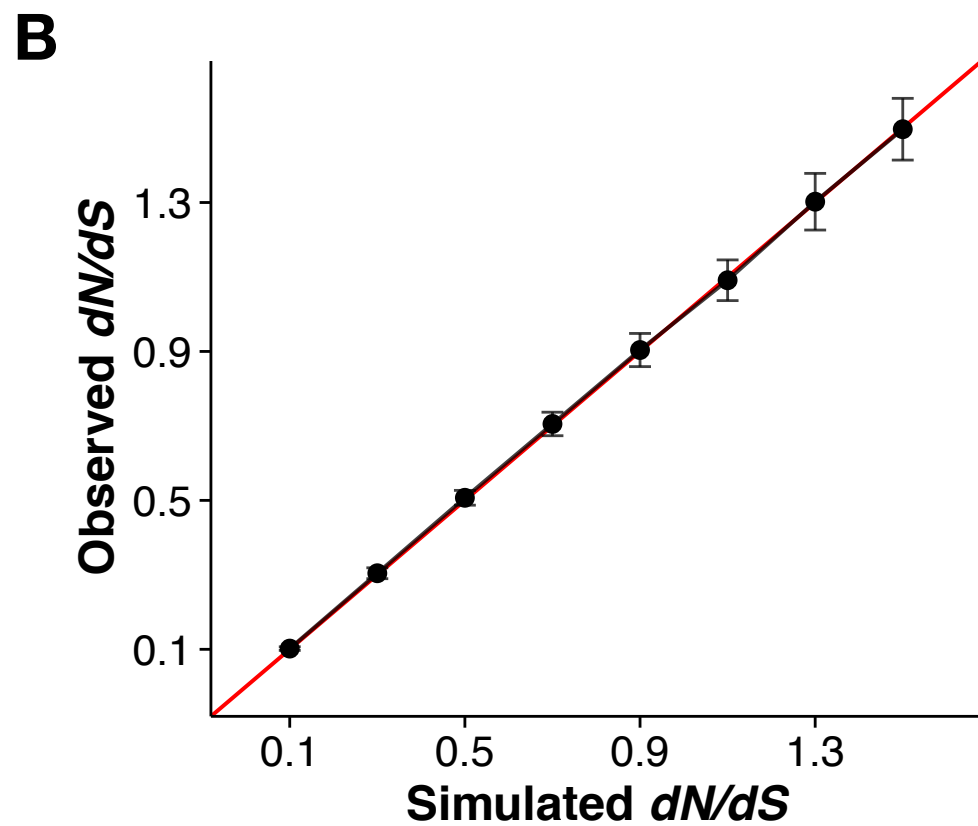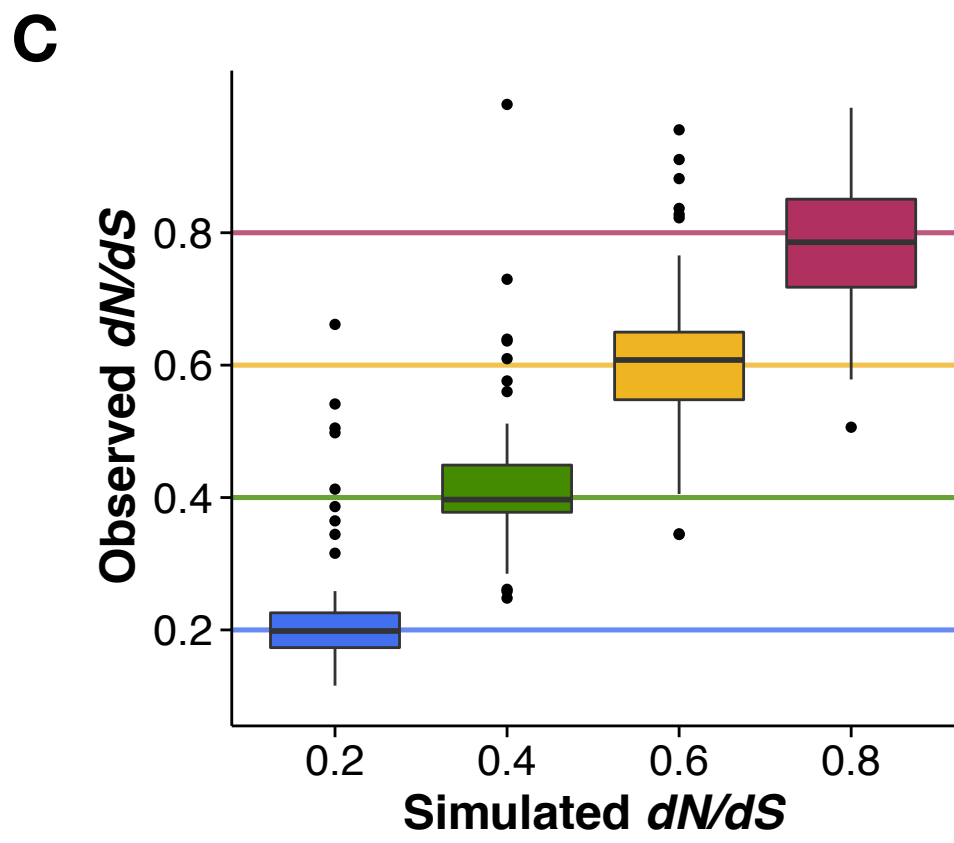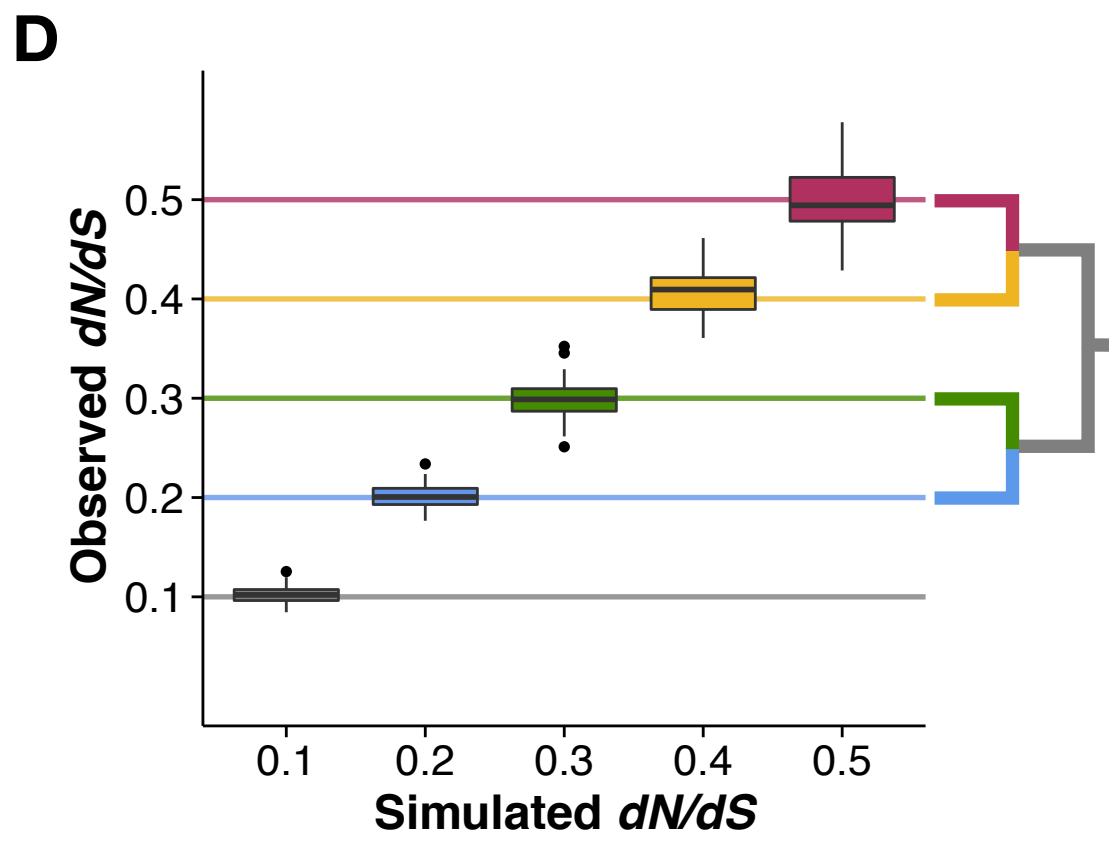

Supplement: S1 File — (ZIP) [file pone.0139047.s001.zip › SI/plot/validated_composite.pdf]

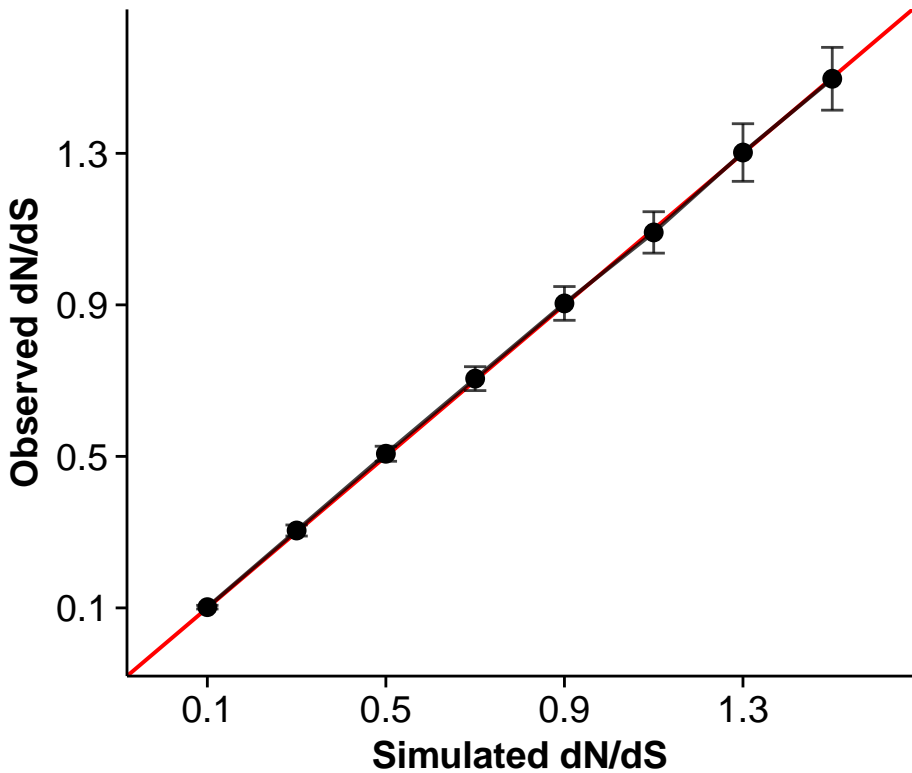

Supplement: S1 File — (ZIP) [file pone.0139047.s001.zip › SI/plot/validated_homogeneous_codon.pdf]

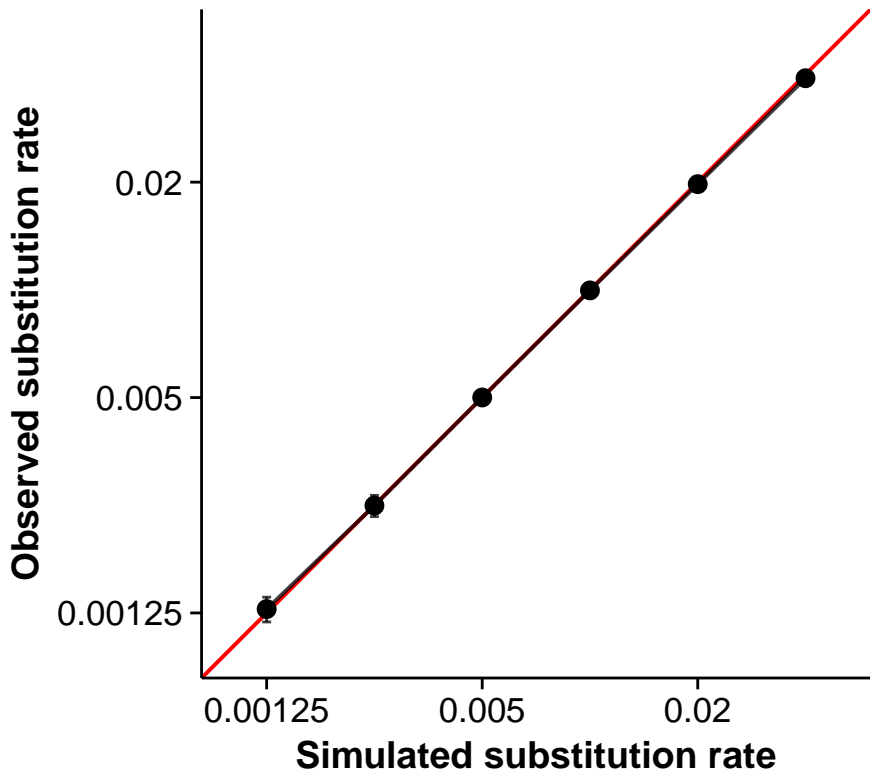

Supplement: S1 File — (ZIP) [file pone.0139047.s001.zip › SI/plot/validated_homogeneous_subrate.pdf]

**Observed dN/dS**

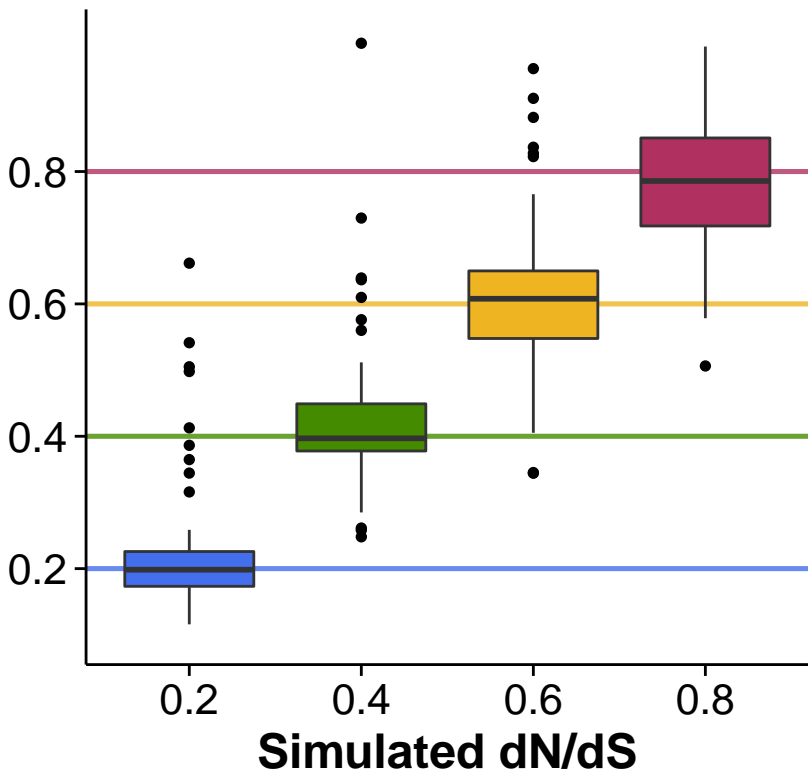

Supplement: S1 File — (ZIP) [file pone.0139047.s001.zip › SI/plot/validated_sitehet.pdf]
